# Anatomy of terminal moraine segments and implied lake stability on Ngozumpa Glacier, Nepal, from electrical resistivity tomography (ERT)

**DOI:** 10.1038/srep46766

**Published:** 2017-04-20

**Authors:** Sarah S. Thompson, Bernd Kulessa, Douglas I. Benn, Jordan R. Mertes

**Affiliations:** 1 College of Science, Swansea University, Singleton Park, Swansea, SA2 8PP, Wales, UK; 2 Department of Geology, University Centre in Svalbard, 9171 Longyearbyen, Norway; 3 School of Geography and Geosciences, University of St Andrews, UK; 4 Geological and Mining Engineering and Sciences, Michigan Technological University, 630 Dow Environmental Sciences, 1400 Townsend Dr, Houghton, MI 49930, USA

## Abstract

Moraine-dammed lakes at debris-covered glaciers are becoming increasingly common and pose significant outburst flood hazards if the dam is breached. While moraine subsurface structure and internal processes are likely to influence dam stability, only few sites have so far been investigated. We conducted electrical resistivity tomography (ERT) surveys at two sites on the terminal moraine complex of the Ngozumpa Glacier, Nepal, to aid assessment of future terminus stability. The resistivity signature of glacier ice at the site (100–15 kΩ m) is more consistent with values measured from cold glacier ice and while this may be feasible, uncertainties in the data inversion introduce ambiguity to this thermal interpretation. However, the ERT data does provide a significant improvement to our knowledge of the subsurface characteristics at these sites, clearly showing the presence (or absence) of glacier ice. Our interpretation is that of a highly complex latero-terminal moraine, resulting from interaction between previous glacier advance, recession and outburst flooding. If the base-level Spillway Lake continues to expand to a fully formed moraine-dammed glacial lake, the degradation of the ice core could have implications for glacial lake outburst risk.

In recent years, the fate of Himalayan glaciers has been hotly debated owing to their role in future water resource scenarios of the Indian Sub-continent^1^, and the impact of associated glacial lake outburst floods (GLOFs)^2^. The debate highlights the disparate nature of predictions of the future of Himalayan glaciers, and the significant gaps in understanding^2^,^3^. While there have been considerable advances in understanding through remote sensing techniques^3^,^4^,^5^,^6^,^7^,^8^,^9^,^10^ and numerical modelling approaches^11^,^12^,^13^, application and interpretations are often hampered by a lack of *in situ* measurements in the Himalayas^2^,^14^,^15^,^16^. Remote locations, high altitude and difficult terrain combine to make *in situ* measurements problematic and therefore rare.

Throughout the Himalayas, potentially hazardous moraine-dammed glacial lakes are becoming increasingly common as a consequence of climatically driven glacier recession^17^,^18^. In the Dudh Koshi region of the Nepal Himalayas, between 1960 and 2000, 11 supraglacial lakes were observed to transition to moraine-dammed lakes^19^, and similar patterns have been observed elsewhere^20^. In addition to the moraine-dammed lakes currently in existence, it is predicted that many more will form in the coming decades as more glaciers cross the threshold required for rapid lake expansion^15^. Susceptible glaciers include those with long, low-gradient ablation zones, such as the Khumbu and Ngozumpa Glaciers in Nepal, where lakes could ultimately attain lengths of several kilometres^15^,^21^. Large moraine-dammed lakes can drain catastrophically if the moraine dam is breached but, can also significantly increase glacial ablation and long-term glacier retreat rates^15^,^21^,^22^,^23^. Very little is known about subsurface structure or internal processes of the moraine dam, which are factors likely to influence dam evolution and stability. A number of processes have been observed to contribute to the degradation of terminal or lateral moraines, such as seepage erosion, and degradation of an ice-cored moraine dam^24^,^25^.

Subsurface investigations traditionally rely on coring and borehole-based surveys, but the intrusive nature of such techniques can lead to artificial modifications of subsurface properties and processes, and surveys are also expensive and logistically difficult in many glacial environments^26^,^27^. More recently, electrical geophysical techniques have been used with increasing frequency and success in both glacial and hydrological investigations^9^,^28^,^29^,^30^,^31^,^32^,^33^,^34^,^35^. Such techniques can provide cost-effective and minimally intrusive complements to more conventional access-hole surveys, facilitating the characterization of subsurface hydrological properties and processes beyond the point scale and thus extending spatial coverage.

Electrical resistivity tomography (ERT) images the DC bulk electrical resistivity (the inverse of bulk electrical conductivity) of the subsurface in 2-D or 3-D^36^. In most geological media, excluding natural ice, bulk resistivity depends principally on the porosity and the saturation and electrical conductivity of the pore waters^36^,^37^. ERT techniques are accordingly well established for hydrological and permafrost investigations, detecting and delineating areas of ice and frozen ground and, changes in the lithological and hydrological properties of the subsurface^26^,^33^,^38^,^39^,^40^. A number of investigations have also applied ERT to delineate internal morainic structures^41^ and identify the presence of ice in glacial moraines^42^,^43^.

Here we report on a series of surface ERT experiments on the terminal moraine complex of the Ngozumpa Glacier, Nepal, to investigate subsurface debris-ice characteristics and aid future assessments of terminus stability. Ngozumpa Glacier is located in the Eastern Himalaya (Fig. 1), an area of significant topographic relief that is climatically dominated by the South Asian monsoon^44^. All glaciers in the region are summer accumulation types and considered highly sensitive to global warming^15^. Ngozumpa Glacier is typical of the many large debris-covered glaciers in the region. The accumulation areas are located on the upper slopes of Cho Oyu (8188 m asl) and Gyachung Kang (7922 m asl) and extensive, precipitous exposed rock faces surround much of the area, resulting in glacier accumulation primarily by snow avalanching. The glacier ablation area extends down to ~4650 m asl, the lower 15 km of which is debris covered, and the lower ~7 km is considered to be stagnant^9^,^45^. The glacier is located in a permafrost region^46^ the lower limit of which is estimated to be >5400 m asl^47^. In the early 1990s, a small base-level lake was identified in the terminal zone of the glacier^21^. By 2009, the lake had reached an area of >300,000 m^2^, with a volume of >2.2 million m^3^ 34. The lake self-perpetuates growth while the spillway, through the western lateral moraine, remains at the same elevation^21^,^34^.

Surface ERT experiments were acquired at (A) a partially healed breach through the western lateral moraine, (B) an area of moraine ridges and hummocky topography on the terminal moraine and, (C) the debris covered glacier surface ~1 km from the terminus (Fig. 1). Site A was identified as an area of interest, located in a breach through the western lateral moraine, likely to be a result of an outburst flood (timing unknown). The breach provides the lowest topographic point on the western side of the latero-terminal moraine complex. The presence of a series of re-advance moraines indicates the breach has been partially healed by moraine-building during subsequent ice-advances (Fig. 1). The structural integrity of the superimposed moraines is unknown, as is the current margin of glacial ice under the debris cover. Nine profiles were collected at Site A (Fig. 1), to ascertain whether the furthest re-advance moraine is ice cored and thus whether the current ice margin extends through the breach. Site B was located towards the centre of the terminal moraine in an area dominated by a lobe-shaped section extending down valley. The area is characterized by multiple traverse, discontinuous ridges and hummocky terrain, and it is difficult to distinguish moraine depositional features from glacier recessional features in the area. The site was chosen to determine whether the surface morphology reflects relatively stable and consolidated moraines, or less stable debris covered glacial ice undergoing differential melting. Site C was located close to the glacier centre line on the debris-covered glacier surface to allow the characterisation of a debris-covered glacier ice resistivity signature and aid interpretation at Sites A and B. At this location, the presence of ice was confirmed and the debris-ice interface located (by digging through the debris) 1.3 m from the surface at a point along the profile (see Methods).

## Results and Interpretation

### ERT results

**Figure 1:**
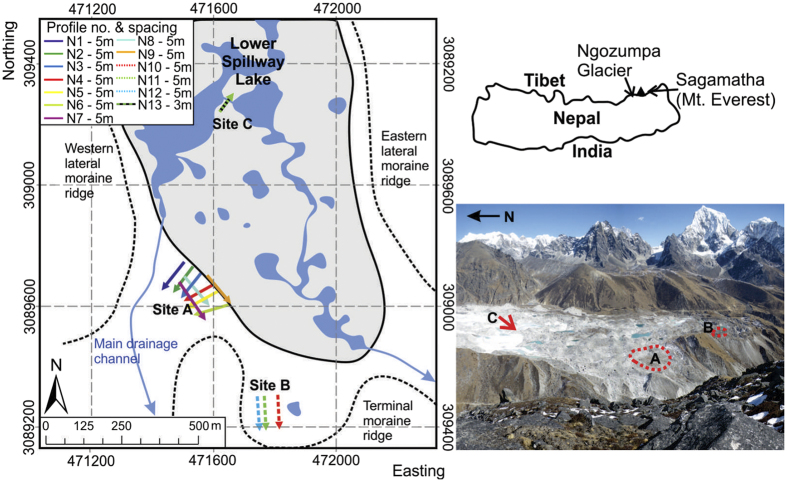
The terminus region of the Ngozumpa Glacier, Nepal, located west of Sagarmatha in the Khumbu Himal region of the Himalayas (location insert not to scale). The map illustrates the main features of the glacier terminus including the base level Spillway Lake and a number of perched ponds, and the three ERT surveys sites, A, B, and C. Coordinates: WGS84 UTM Zone 45N (Maps produced by authors, site map generated from GeoEye-1 image from 9^th^ June 2010 in ArcGIS 10.4 http://desktop.arcgis.com/en/).

**Figure 2:**
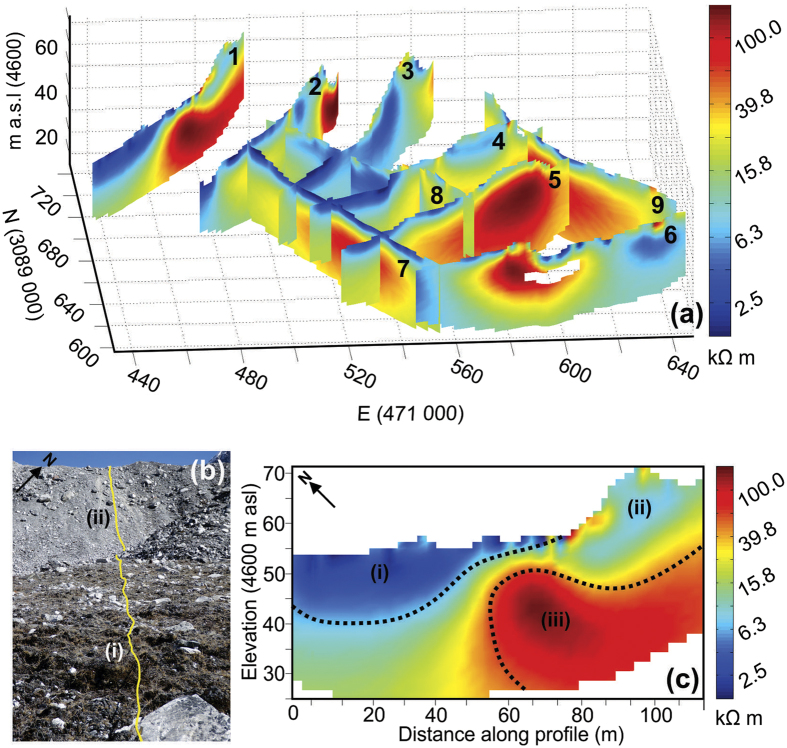
(**a**) 2-D inversion results of the nine ERT profiles acquired at Site A plotted in a 3-D grid; Profiles are numbered 1–9, matching descriptions are given in the text. The absence of data in the centre of profile 6 in Site A was a result of the application of the DOI index (see Methods). Coordinates: WGS84 UTM Zone 45. (**b**) Site A, Profile 1 data acquisition (resistivity cable marked in yellow), highlighting the differing surface characteristics, (i) unsaturated debris/moraine comprised of finer sediment or soil matrix, the more stable regions of which are vegetated and (ii) unsaturated debris/moraine of a coarser, dryer appearance at the surface. (**c**) Site A profile 1 resistivity inversion output. The differing surface characteristics identified at the site are associated with different resistivity signatures in the profile, (i) very low resistivity signature (1–2 kΩ m) and (ii) low resistivity signature (5–10 kΩ m) with the addition of (iii) an area of very high resistivity (>100 kΩ m) below the surface.

**Figure 3:**
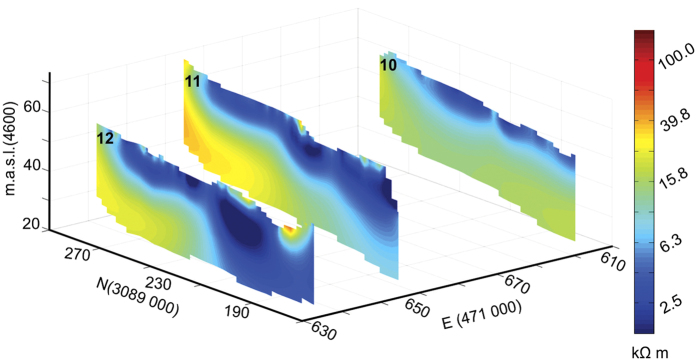
2-D inversion results for the three ERT profiles acquired at site B, plotted in a 3-D grid. A low resistivity signature (1–5 kΩ m) dominates the surface layers, with a moderate signature, 10–20 kΩ m at depth. The high signature (>100 kΩ m) present at sites A and C is absent from these three profiles. Coordinates: WGS84 UTM Zone 45.

**Figure 4:**
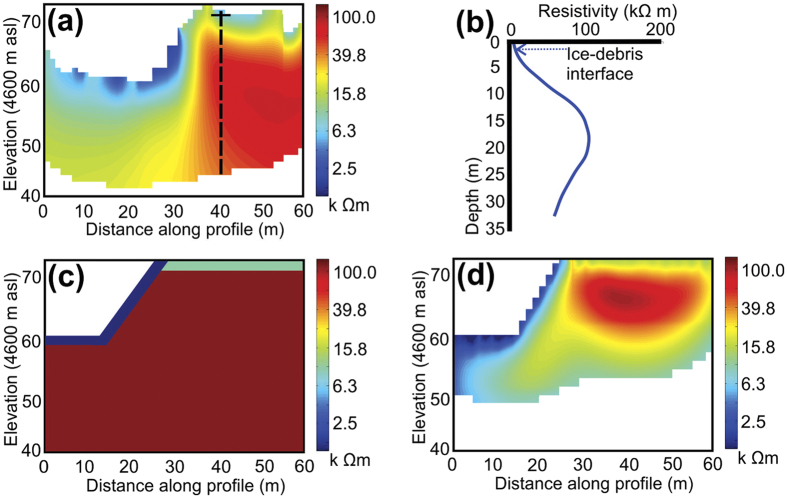
Profile 13; (**a**) The 2-D inversion results for profile 13, site C. Depth to the debris-ice interface (solid line) was determined at 1.3 m (at the intersection with dashed line). (**b**) Resistivity values down a column of profile 13 where the depth to debris-ice interface was measured (dashed line in (**a**)). (**c**) The synthetic model of subsurface properties, based on profile 13, for synthetic resistivity data generation. (**d**) 2-D synthetic resistivity inversion results with the application of the DOI index (see equation 4).

At all three sites on the Ngozumpa terminus (Fig. 1), bulk resistivities ranged from ~1 kΩ m to >100 kΩ m (Figs 2, 3 and 4a). At sites A and C two dominant resistivity signatures were identified; low values (<10 kΩ m) occurring predominantly in the surface layers (0–10 m) and, areas of high resistivity (>100 kΩ m) at depth (>10 m) (Figs 2 and 4a). Site B exhibits the low resistivity signature (1–10 kΩ m), which extends to depth in profile 12 and higher values (>100 kΩ m) are absent from these three profiles (Fig. 3). Outside these two classifications, intermediate resistivity signatures show a broad, gradual transition from ~10 kΩ m to 80 kΩ m (Figs 2 and 3).

The lower resistivity values (~1–10 kΩ m), occurring in the surface layer of all profiles (Figs 2 and 3) are consistent with unsaturated and unfrozen sediments (as exposed at the surface)^33^,^36^,^48^. Given the variation in surface conditions at the site (e.g. Fig. 2b), the lowest resistivity values (1–5 kΩ m) could represent areas of unsaturated debris/moraine comprised of finer sediment or soil matrix, the more stable regions of which are vegetated ((i) in Fig. 2b,c). These finer soil-like matrices encourage storage of moisture to a greater degree than the coarse glacier debris, thus reducing bulk resistivity. Where higher resistivity values are found in the surface layer (5–10 kΩ m), they are consistent with those expected for dry morainic material (5–10 kΩ m)^36^. At the surface, such values occur in areas where the surface debris appears coarser and dryer ((ii) in Fig. 2b,c).

In all profiles the transitions between areas of different resistances are smooth (Figs 2, 3 and 4a), even with the inversion roughness coefficients set to emphasise a blocky rather than smooth inversion (see Methods). While a gradual transition from surface debris, through ice-rich debris to glacier ice is feasible, ground-truth data in profile 13 revealed the ice-debris interface to be sharp and well defined; unfrozen debris overlying clean glacier ice. The well-defined transition is not however reflected in the resistivity signature at this location and, the gradual transition observed is likely an artificial effect (Fig. 4a,b).

### Sub-debris ice interpretation

When liquid water in the ground freezes, the mechanism of electrical conduction remains dependant principally on the porosity and saturation and electrical conductivity of the pore waters until the size of pore space decreases to less than a few micrometres^36^,^37^. Interstitial ice then begins to play an increasingly important role in electrical conduction^49^, and a marked increase in resistivity occurs^50^. The mechanisms of electrical conduction in natural ice, such as that contained in polar ice sheets, mountain glaciers or frozen ground, are still not fully understood^31^. Increasing evidence now suggests that such conduction is dominated by the migration of charged protonic point defects in single ice crystals in accordance with Jaccard theory, irrespective of whether the ice is cold or temperate or whether situated in polar ice sheets, mountain glaciers or frozen ground^49^,^51^.

*In situ* measurement of bulk resistivity of ice typically varies over several orders of magnitude, dependant on the temperature and character of the ice. Bulk resistivities for perennially frozen ground and Alpine rock glaciers have been reported in the range of 10–100 kΩ m^52^,^53^,^54^ and values for cold glacier ice reported in the region of 100 kΩ m^55^,^56^,^57^. For temperate glacier ice values are usually reported to be much higher, at > >1000 kΩ m, even under extensive debris covers^48^,^58^. The high resistivity values (>100 kΩ m) at Site A (Fig. 2) exceed those characteristic of regional geological materials (with the exception of bedrock) and, are consistent with values measured from Alpine rock glaciers and cold glacier ice^52^,^53^,^55^,^57^,^59^.

**Figure 5:**
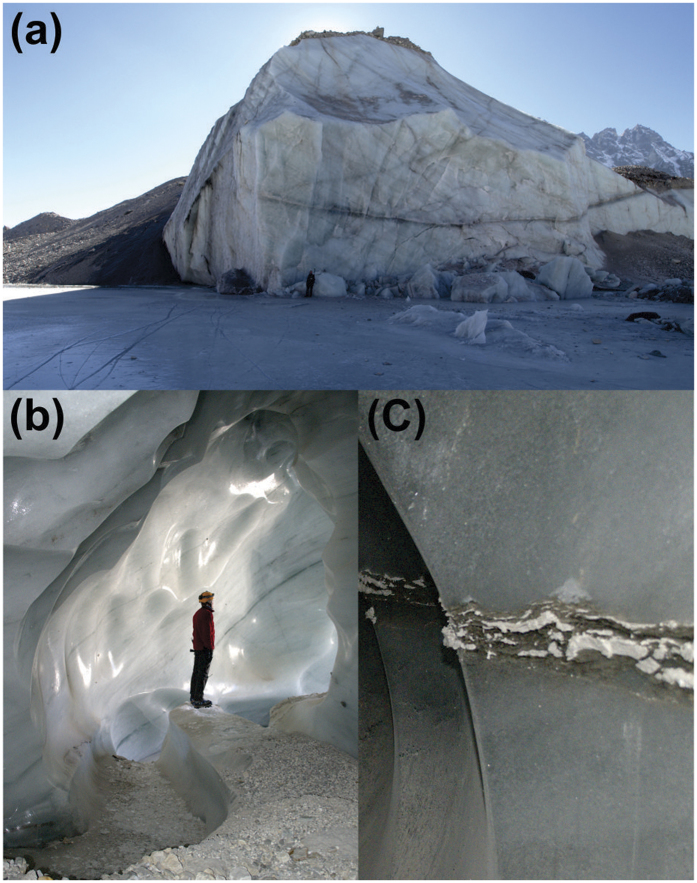
(**a**) Sub-debris ice exposed in an ice cliff bordering Spillway Lake in the lower ablation area of Ngozumpa Glacier. Ice fractures are consistent with metamorphosis during transport though ice falls and debris bands are discrete. (**b**) Glacier ice in englacial conduits in the lower ablation area of Ngozumpa Glacier has very low debris concentrations. (**c**) Debris bands exposed in the walls of englacial conduits in the lower ablation area of Ngozumpa Glacier are generally thin and discrete.

The internal character and structure of Alpine-style rock glaciers has been characterised at numerous sites, the majority of which report combinations of glacier ice, ice lenses and sediment and debris cemented by interstitial ice^52^,^53^,^60^,^61^. Feasibly rock glaciers and small debris-covered glaciers in steep terrain could contain a mixture of avalanche snow, heavily broken avalanche ice and rock-fall debris, (re-) frozen under permafrost conditions. The mixture has been previously termed ‘congelation ice’^52^. However, ‘congelation ice’ does not describe the nature of sub-debris ice for the Ngozumpa Glacier or other large Himalayan debris-covered glaciers. The ice in the terminal zone of Ngozumpa Glacier has been transported at least 10 km from any possible source area, and at present most of the ice originates in snowfields or snow-rich avalanche cones^15^. In numerous exposures in the lower ablation area of the glacier (and in the neighbouring Khumbu Glacier) ice is seen to have typically low debris concentrations with discrete debris bands (Fig. 5)^9^,^15^,^62^,^63^. Ice properties are also consistent with significant metamorphosis during transport (Fig. 5)^62^. Both observations and theoretical considerations indicate that ‘permafrost-frozen avalanche material’ is not present near the terminus of Ngozumpa and due to the comparable signature at Profile 13, where ground truth exists (Fig. 4a), the high resistivity values (>100 kΩ m) at Site A (Fig. 2) are interpreted as glacier ice (see Methods).

Despite significant uncertainty in the actual electrical conduction mechanism, the resistivity signature of glacier ice at Ngozumpa Glacier is a magnitude lower than values typically recorded for temperate glacial ice^31^,^48^,^64^,^65^. Very little is known about the thermal regime of large Himalayan debris-covered glaciers. Measurements in shallow boreholes in the upper ablation area of the neighbouring Khumbu Glacier in the 1970s, reported a 16 m thick cold layer overlying temperate ice^66^. It has been suggested the Khumbu Glacier is likely polythermal^63^; accumulation occurs in extremely cold, high altitude environments facilitating the formation of cold ice^44^. Considering the high-altitude accumulation area, the following section discusses whether cold ice can feasibly be present in the lower ablation area at Ngozumpa Glacier.

Evidence from the European Alps suggests that very few mountain glaciers are temperature thoughout^67^. The advection dominant thermal structure of the Groner Glacier in the Swiss Alps has been extensively studied, revealing a central core of cold ice (≥−2.5 °C), advected from the high altitude accumulation area through to the glacier terminus^67^,^68^. The work illustrates that cold ice can be transported over long distances by the movement of ice in an Alpine glacier, even within zones of temperate surface temperatures. On the Ngozumpa Glacier, the accumulation areas are likely composed of cold ice owing to the high altitude (7000 to >8000 m asl) and very low average annual temperatures (<−10 °C at ~7000 m asl)^44^. Borehole ice temperature at 6325 m a.s.l on the nearby East Rongbuk Glacier was measured at −9.6 °C at a depth of 20 m^69^. The ice temperature will increase down-glacier, the primary influences being; strain heating, surface conduction, and basal geothermal heat flux. Strain heating may provide a significant source of heat energy but will be confined to faster-flowing icefalls, located in the accumulation area and upper reaches of the ablation area. In the lower 7 km of the ablation area, strain heating will be minimal since glacier-ice is stagnant and thinning^9^,^45^. The contribution to heat energy from surface conduction, in the accumulation area, will be minimal or negative, in this region, even at ~5000 m asl, temperatures above 0 °C are only recorded for ~25% of days per year^44^. In the lower ablation area, the debris cover becomes spatially-continuous (with the exception of exposed ice cliffs around lakes or ponds), exceeding 2–3 m in thickness near the terminus^21^. Once debris thickness is >1 m, the glacier-ice surface is effectively insulated from incoming radiation^11^,^70^.

Geothermal heat-flux is poorly known in the Khumbu Himal, but better constrained in the Himalayan Geothermal Belt (HGB) where heat fluxes range between 60–90 mW / m^2^, the continental global average is ~65 mW / m^2^ 71. A 1D heat flux model was used to investigate geothermal heat flux on the East Rongbuk glacier, based on bore hole ice temperature, results suggest a geothermal heat-flux as low as 18.5 mW/m^2^ 12,69. In the Khumbu Himal, geothermal heat-flux is expected to be low (<60 mW/m^2^) as indicated by a lack of hot springs, a feature synonymous with higher geothermal heat-flux elsewhere in the HGB^71^. Given the similar altitude ranges and close proximity, it is likely that the Ngozumpa Glacier may exhibit a similar thermal regime to that modelled on the East Rongbok Glacier^12^. The resistivity values (>100–150 kΩ m) more consistent with cold glacial ice suggest that there is a possibility glacial ice imaged by ERT could be cold; thus, out of equilibrium with local climate. While the near surface layer (≥10 m) is affected by cold winter conditions, ice-resistivities between this layer and depths of ~30–35 m are remarkably homogenous (e.g., Fig. 2c). Seasonal temperature variations are unlikely to penetrate below 20 m in clean-ice glaciers^72^, a depth limit that is even more reduced in debris-covered glacier ice since debris has a lower thermal conductivity than ice (0.5–1.7 W/m/K^11^ and 2.1 W/m/K^72^ respectively). The lack of evidence of seepage or icings at the glacier terminus also suggests that glacier drainage is concentrated englacially and supraglacially. If the basal component is as it appears, negligible, there may be little or no liquid water at the bed.

### Uncertainties

Two main effects introduce significant uncertainty into the inversion outcomes and thus, the interpretation of the resistivity signatures at Ngozumpa Glacier. Firstly, all ERT data are collected along 2-D profiles and then inverted assuming that the medium is effectively invariant in the third direction. As a consequence, the 2-D representation of an actual 3-D distribution of electrical subsurface properties leads to an uncertainty in the inverted image which is not generated by data error or noise^73^. The effect will be exacerbated in areas of significant topography^74^. Secondly, the high contrast in resistivity within the profiles is increasing what is known as the inherent equivalence problem^75^. When a relatively thin conductive (or resistive) layer is encountered, the inversion resolves the product of conductivity, thickness or resistivity and thickness, rather than the exact values of conductivity and thickness separately. Additionally, if a mid-layer has physical properties between those of the overlying and underlying layers, the presence of the layer is suppressed in the data. Resolving such a layer is generally very difficult even when including a priori information about the presence of such a layer^76^.

As little is known about the subsurface characteristics at Ngozumpa, negating the use of a priori information, we develop a synthetic resistivity data model (See Methods). The model, based on the inversion results and ground truthing from profile 13 (location Fig. 1), allows investigation of the uncertainty in the reported resistivity signatures. The generated synthetic model consists of a debris surface layer (~1.3 m), of 1 kΩ m to the left half of the profile, where debris at the surface of profile 13 appears fine and unsaturated, and ~10 kΩ m to the right, where debris at the surface appears blocky and dry. Beneath the debris surface layer, a glacier ice signature (~100 kΩ m) extends to the depth of the profile (Fig. 4c). The synthetic model was adapted and the process repeated until both the synthetic and real data inversions produce similar results (Fig. 4). The final synthetic data model required a glacier ice layer value of 150 kΩ m to produce inverted resistivity values similar to those of profile 13 at site C (Fig. 4a,d).

The inverted synthetic resistivity model, like profile 13, shows evidence of resistivity signature suppression, likely a result of the equivalence problem. The subsurface glacial ice layer (150 kΩ m) extends along the full length of the synthetic model profile at depth (Fig. 4c), under the assumption that this is the case for profile 13, located toward the glacier centreline. However, the left-hand side of both inverted profile 13 and the synthetic model (Fig. 4c) exhibits a reduced resistivity signature (10–25 kΩ m) beneath the lower resistivity (~1 kΩ m) surface layer (Fig. 4a,d). The results indicate the expression of the more highly resistive glacier ice is suppressed by the overlying less resistive surface debris layer.

### Glaciological implications

The profiles acquired at site A, near the latero-terminal moraine (1–9, location Fig. 1), combined with the moraine morphology and sediment characteristics visible at the surface, illustrate the compound, structurally complex nature of the moraine system. Close to the current main drainage channel through the western lateral moraine, an ice-cored moraine ridge with relatively unconsolidated debris cover, has partially healed a former breach through the moraine (Site A-Figs 1 and 2). The ice margin now appears to extend through the original breach, beyond the line of the latero-teminal moraine, however; the thickness of the glacier ice cannot be determined beyond the depth of the ERT profiles (~35 m allowing for pronounced surface topography) (Fig. 2). The ice-cored re-advance moraine appears to be superimposed over older morainic material, which at the surface has a soil-like matrix and is partially vegetated.

At Site B there is an absence of the higher resistivity signature (>100 kΩ m) (Fig. 3) suggesting that this location is currently beyond the detectable glacier ice limits, at least to a depth of ~30–35 m. The absence could indicate that the observed ridged, hummocky surface topography (Site B, Fig. 1) reflects debris deposition and moraine building over alternating minor advances and retreats (as proposed in the Introduction). However, the synthetic modelling of profile 13 (Fig. 4d) illustrating a significant suppression of the bulk resistivity signature at depth with a more conductive surface layer, adds uncertainty to this interpretation. At this site B the partially vegetated, soil-like surface layer will hold more moisture, increasing conductivity and could extend to a depth of several meters. It is possible that this is sufficient to effectively suppress the signature of ice at depth in the inverted resistivity profiles (Fig. 3).

The presence of glacier ice within the breach moraine complex in the western lateral moraine (Site A) may have consequences for the stability of a future moraine dam. Despite ambiguity in the thermal interpretation of the high resistivity values (>100 kΩ m), the identification of the debris-ice interface in profile 13 confirms that this signature is indicative of glacier ice at this site. If the glacier ice identified in the latero-terminal moraine ridge is indeed cold (>30 m), it suggests the ice core is currently well insulated. Seepage and piping are phenomena known to undermine the longer-term stability of moraine dams^77^, but a lack of liquid water close to the terminal moraine suggests little internal ice degradation is occurring. However, in the longer term the distinction of cold or temperate ice would have little direct impact upon rates of ice-melt. If the base-level Spillway Lake continues to expand to a fully formed moraine-dammed glacial lake, the re-advance moraine will likely be an integral part of the moraine dam and the degradation of the ice core could have serious implications for glacial lake outburst risk^78^. Thus, for moraine dam investigation and glacial lake hazard prediction at this site, it is the ability to detect the presence or absence of glacial ice that is paramount and the ERT data does provide a significant improvement to our knowledge of the subsurface characteristics at these sites.

### Synthesis and Future Work

ERT surveys at Ngozumpa Glacier reveal a highly complex latero-terminal moraine, resulting from interaction between previous glacier advance, recession and outburst flooding. The work highlights the compound nature of debris-covered glacier moraines and the complex range of processes that can occur within them. The presence of ice within the re-advance moraine could pose an increased risk of outburst flood if the base-level Spillway Lake continues to expand to a fully formed moraine-dammed glacial lake. Better prediction of longer-term moraine-dam stability necessitates investigation of the entire moraine structure. At this stage, focusing efforts in one area alone cannot give a reliable prediction of subsurface characteristics in another, even where surface morphology is similar. Further, it is not currently possible to determine the longer-term stability of a moraine dam from remote sensing or by analogy to other moraine dam sites.

The ERT technique clearly identified glacier ice in the re-advance moraine at site A, based on the characterisation of the ice signature at site C. However, the limitation of the 2-D survey and inversion, combined with the surface topography and high resistivity contrasts causing equivalence, posed significant uncertainty in interpretation. Such uncertainty in interpretation is clearly illustrated by the result of the inversion of the synthetic data set (Fig. 4b,c), based on the result of the inversion of data at site C (Fig. 4a). The synthetic data model has a sharp debris-ice interface, with sub-surface ice extending the length of the profile (Fig. 4c) but the inversion results of the data set show both a gradual interface and a resistivity signature of glacier ice is only evident to the right side of the profile (Fig. 4d). The same phenomena could be supressing the signature from ice at depth in the profiles at Site B where the surface layer may be thick and is more conductive. While the suggestion of cold ice at depth would appear conceptually feasible, these limitations introduce uncertainty in the absolute value of the resistivity magnitude. It is likely that the resistivity signature usually associated with cold ice (>100 k Ω m) is a result of the higher conductive surface layer supressing the signature of the lower ice layer to some extent. In addition, the gradual transition from surface debris to glacier ice observed in the profiles at sites A and C is unlikely to be realistic, further suggesting that while the technique is successful in identifying the presence (or absence) of glacier ice beneath the debris, the spatial extent and thermal character of the ice remains unknown.

Further investigation is urgently required to better constrain the results and interpretation of the ERT surveys in this environment and, to determine the spatial and temporal extent of their applicability. The highly variable surface topography and significant resistivity contrasts, render a 2-D representation of the 3-D distribution of electrical subsurface properties subject to uncertainty, thus future surveys should be collected and inverted in 3-D. In addition, the effect of a thick, conductive debris layer must be quantified to allow further use of the technique for moraine dam applications. Laboratory experiments investigating the effect of a debris layer of differing characteristics on the resistivity signature from underlying glacier could reduce the uncertainty in the interpretation of the presence or absence of ice at different sites. If a more robust characterisation of the resistivity signatures of debris-covered ice could be developed, the large difference in the resistivity signature between cold and temperate ice could be used to aid investigation the thermal regime of debris-covered glacial ice.

Geophysical surveys are typically constrained by some prior knowledge of the subsurface; debris thickness and morphology data, as well as ice temperature measurements are key to further investigation. However, the high altitude, isolated locations of glaciers such as the Ngozumpa, provide significant challenges to such an approach. A common approach in Alpine glacier and permafrost applications has been to employ two or more geophysical techniques at a single survey location^32^,^78^,^79^,^80^,^81^. On Himalayan debris-covered glaciers multi-technique geophysical surveys can be logistically and physically difficult and therefore are rare. Various geophysical methods used to investigate the subsurface utilize different physical principles; while ERT exploits the variable resistance of different materials to conduct an electrical current, refraction seismic techniques exploit the elasticity and density properties of different materials, and ground penetrating radar (GPR) utilises the attenuation and reflection of an electromagnetic signature at dielectric boundaries in the subsurface. Combined application and interpretation of two or more techniques has shown to significantly reduce ambiguity in output and inversion interpretation, particularly with regard to resistivity signature suppression^32^,^79^. GPR is a well-established technique for clean ice glaciers and, use in combination with ERT to identify the boundaries between different resistive layers, is well established in groundwater research^81^. Refraction seismic techniques have been little used on debris-covered glaciers, but the individual application of both ERT and refraction seismic techniques to rock glaciers and to a lesser extent, glacial moraines^27^,^41^,^82^ illustrates significant potential for their combined use on Himalayan debris-covered glaciers and moraine dams. There is a sharp increase in seismic wave velocities at the freezing point (average wave velocities for glacial ice and glacial debris (3.6–3.8 km s^−1^ and 0.5–2.6 km s^−1^ respectively)^27^ allowing detection of glacial ice^83^,^84^.

### Methodology

In November 2010, 2-D ERT surveys were carried out at three locations on the Ngozumpa Glacier with the aim of identifying the ice margin at two key locations (Fig. 1). All ERT data were collected using an IRIS Syscal-Pro 24-node imaging system with stainless-steel electrodes. The standard Wenner Alpha acquisition geometry was used as it is known to yield particularly stable results in resistive, heterogeneous settings such as ours^33^,^48^. Array choice is a particularly important consideration for surveys of significant topography and resistive heterogeneity, characteristic of many debris-covered glacier and moraine sites. The Wenner array has good depth penetration and resolution as well as a high signature to noise ratio but it is less sensitive to horizontal changes in resistivity^85^. The Schlumberger array is similar, except that the spacing between the current electrodes is unequal to that of the potential electrodes. It has similarly good depth penetration and slightly better spatial resolution. However, both Wenner and Schlumberger array configurations tend to pick up more noise than other common arrays when using the reciprocal configuration^85^. A frequently used alternative is the dipole-dipole array which has very good horizontal resolution, and reciprocal measurements work well. However, the signature to noise ratio is often poor and, sensitivity to errors in the spacing between electrodes is high, a serious consideration in complex terrain. More recently a hybrid configuration between the Wenner and Schlumberger arrays was developed^86^, with slightly better and wider horizontal coverage than the Wenner array and a median depth of investigation ~10% larger. The Wenner–Schlumberger array was employed at the Ngozumpa field but to achieve the improved data coverage, the Wenner–Schlumberger array collects more quadripole measurements (121) than the standard Wenner array (84). Very cold and frequently overcast conditions at the field site during data acquisition, shortened the battery life and reduced the effectiveness of solar power. Under such conditions, we frequently experienced insufficient current injection for the Wenner–Schlumberger measurements. The Wenner array proved more successful, due to the shorter data collection time the required injection current was maintained for all measured points. Therefore, for surveys of significant topography and resistive heterogeneity in remote locations where battery life is a consideration, the Wenner array remains the most suitable array geometry.

To minimize uncertainty caused by ground heterogeneity and significant local topography, rigorous error analysis was adopted from our previous work^48^. Both repeat and reciprocal data sets were acquired in all cases to quantify acquisition errors, anomalies (>2 σ of the mean) and measurements with an error >5% were removed from each data set. Linear regression was applied to the two data sets to allow the identification of points with disparate normal and reciprocal measurements. 95% confidence bands were applied to the regression line, and any data points that fell outside of these bands and were removed^48^.

All resistivity data were inverted using the finite-difference code DCIP2D^87^,^88^ using 7 data levels, with an average of 12 iterations required to produce final inverted ERT models that fitted the data to within a 5% root-mean-square error (RMS) error level. The code minimizes a discretized version of the model objective function^87^,


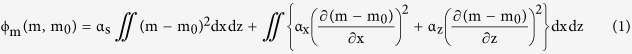


where m and m_0_ are the current and reference models respectively. The constant α_s_ (damping factor) controls the closeness between the current model and the reference model, while the constants α_x_ and α_z_ control the model roughness in the horizontal and vertical directions respectively (default values of 0.001, 1 and 1 were used in all data inversions). Minimization is achieved iteratively by updating m with respect to m_0_ based on the misfit functional, ϕ_d_


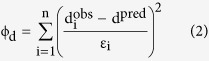


where 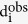 and d ^pred^ are the measured and computed data respectively, and ε_i_ are the measurement errors. In practice this is not straight forward, the inverse problem is non-unique; an infinite number of models will fit the data to a required level of uncertainty^89^,^90^. In addition to uncertainties already discussed (see Results section Uncertainties), instability inherent in the inversion also means that arbitrarily small errors in the data can still generate arbitrarily large errors in the model^91^,^92^. This problem is further exacerbated with high resistivity contrasts (expected in our data), known to favour the development of inversion artefacts^93^,^94^,^95^. A popular approach is to include a priori information in the inversion by creating initial or reference models^96^,^97^, or as a direct constraint on the inversion. An alternative is to forward model a synthetic resistivity data model^95^,^98^. In contrast to the inverse problem, the forward problem is unique, allowing investigation of the response of the inversion process to a given subsurface structure. Forward modelling is accomplished using the finite difference code DCIPF2D^87^,^88^, which solves


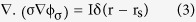


where σ is the electrical conductivity (S/m), ∇ is the gradient operator, I is the strength of the input current (A), and r_s_ is the location of the current source. The potential ϕ_σ_, is the potential due to a single current.

The synthetic data set was based on the same Wenner Alpha array configuration with 24 electrodes at 3 m spacing as the real Profile 13 (location Fig. 1) data collection set up, with the addition of 5% Gaussian random noise. A model including an approximation of surface topography was inverted to assess the spatial variation within the profile (Fig. 4c,d). The inversions were carried out using the same finite-difference code DCIP2D, which was stopped as soon as the data misfit value fell below the noise level (an average of 11 iterations). The resistivity of the lower glacier ice layer of the synthetic model was adapted and the process repeated until both the synthetic and real data inversions produced similar results (Fig. 4).

Considering the high resistivity contrast and the significant topography in the survey areas, a further two complementary reliability checks were carried out on the inverted real data. The aim of which was to eliminate any remaining inversion artefacts and assess the data resolution at depth. First, the depth of investigation (DOI) index was adopted^48^,^95^,^99^ and applied to each profile. This index requires two further inversions to be carried out for each profile, using alternating reference models set to values of one-tenth and ten times the reference model used for the profile data inversion. An increase in damping factor to α_s_ = 0.05 is used to ensure closeness between the reference and iteratively updated current models. The DOI index is then calculated from


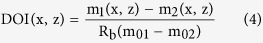


where m_1_(x, z) and m_2_(x, z) are the resistivity values in the two final models, and m_01_ and m_02_ are the values of the corresponding reference models. R_b_ are the resistivity values at the base of the two final models, expected to be equal to, or at least very close to, the corresponding value of the reference model. In areas where the final models are well constrained by the data, and the inversion process considered reliable, the DOI index is close to zero. Any cells with a DOI index greater than 0.2^48^,^95^,^99^ were considered less reliable and thus rejected (Fig. 4a,d).

Secondly, the inverted final resistivity values along the one-dimensional intersection between any two ERT profiles were plotted against each other where possible (locations at Site A, Fig. 1). This was carried out after application of the DOI index, so that the final resistivity values of any two intersecting ERT profiles should be near identical if the inversion process was reliable. At Site A, profiles that intersected near the centres of both profiles, show a significant positive correlation (R^2^ = 0.90–0.98). Where the intersection occurs close to the end of one or both profiles, values are poorly correlated (R^2^ = 0.52–0.63). This is likely to be a result of the lack of sensitivity towards each end of the region beneath the electrodes in the Wenner array configuration. The application of the DOI index does not remove the regions as there is sufficient data to alter the resistivity value from the reference model. The magnitude of the values in these end regions will be subject to greater uncertainty as they are based on fewer measurement points. This effect is likely to be exacerbated at sites, such as Ngozumpa Glacier, where the subsurface can be very heterogeneous. While confidence can be held in the magnitude and broad spatial pattern of resistivity values due to rigorous error quantification, greater uncertainty is present towards the ends of the profiles and in the exact location and nature of resistivity boundaries.

## Additional Information

**How to cite this article**: Thompson, S. S. *et al*. Anatomy of terminal moraine segments and implied lake stability on Ngozumpa Glacier, Nepal, from electrical resistivity tomography (ERT). *Sci. Rep.* **7**, 46766; doi: 10.1038/srep46766 (2017).

**Publisher's note:** Springer Nature remains neutral with regard to jurisdictional claims in published maps and institutional affiliations.
